# Optical coherence tomography features and risk of macular hole formation in the fellow eye

**DOI:** 10.1186/s12886-021-02111-1

**Published:** 2021-09-29

**Authors:** Birger Lindtjørn, Jørgen Krohn, Vegard A. Forsaa

**Affiliations:** 1 Department of Ophthalmology, Stavanger University Hospital, box 8100, N-4068 Stavanger, PO Norway; 2 Department of Clinical Medicine, Section of Ophthalmology, University of Bergen, Bergen, Norway; 3 Department of Ophthalmology, Haukeland University Hospital, Bergen, Norway; 4 Department of Quality and Health Technology, University of Stavanger, Stavanger, Norway

**Keywords:** Bilateral macular holes - epidemiology - macular hole - optical coherence tomography - risk factors - vitreoretinal surgery

## Abstract

**Background:**

To investigate the risk of primary macular hole (MH) in the fellow eye, and to evaluate baseline characteristics and optical coherence tomography (OCT) features that precede MH formation in the fellow eye.

**Methods:**

A retrospective review of 229 patients treated for primary MH at Stavanger University Hospital, Norway, from January 2008 through December 2018. The patients were categorised into two groups according to subsequent development of MH in the fellow eye. The OCT findings of the two groups were compared, and associated risk factors for MH formation assessed.

**Results:**

Twenty cases of bilateral MH were identified. The overall bilateral disease risk was 8.8% (95% CI, 5.8–13.2%). Two patients were previously operated in the fellow eye, six patients presented with bilateral MH, and 12 patients subsequently developed MH in the fellow eye. The risk of subsequent MH development was 5.7% (95% CI, 3.3–9.8%). Although the extent of posterior vitreous detachment (PVD) tended to be more progressed in the bilateral group compared with the unilateral group, the difference was not statistically significant. In the bilateral group, 41.7% had outer retinal defects vs 6.6% in the unilateral group (*p* = 0.001), and 33.3% in the bilateral group had intraretinal pseudocysts vs 10.2% in the unilateral group (*p* = 0.036, not significant after multiple testing correction).

**Conclusion:**

Outer retinal defects and intraretinal pseudocysts are associated with an increased risk of MH formation in the fellow eye, and complete PVD indicates a decreased risk of MH formation.

## Background

The incidence of primary full-thickness macular hole (MH) is 7.9–8.7 eyes per 100,000 population per year [1, 2]. MH predominately occur in the elderly population with a male-to-female ratio around 1:3 [2]. A small percentage of MHs close spontaneously, varying between 4.0 and 11.5% [3]. If left untreated, the MH size increases over time and severely reduces the visual acuity (VA) to less than 20/200 in the majority of cases [4, 5].

The pathogenesis of MH formation is not yet fully understood. However, it is generally accepted that anteroposterior traction at the vitreoretinal interface is a major contributor to the development of MH [6]. Previous studies on the risk of bilateral MH have estimated the risk to be between 7.0 and 16.7% [1, 2, 4, 7–10]. The use of spectral domain optical coherence tomography (SD-OCT) and swept source optical coherence tomography (SS-OCT) enables detection of subtle retinal abnormalities. Some studies have investigated changes at the vitreoretinal interface and showed that foveal or complete posterior vitreous detachment (PVD) indicates a low risk of MH formation [7, 11–13]. Studies on retinal abnormalities in fellow eyes have revealed certain structural changes that are associated with an increased risk of MH formation [7, 11, 14, 15].

Patients with MH often ask for information about the risk of developing MH in their fellow eye, and selected patients with a predicted high risk may require regular follow-up examinations and early surgical intervention. This present study sought to provide some answers to these questions based on the evaluation of retinal morphological changes in the fellow eye of patients with MH. The aims of the study were to determine the risk of developing bilateral MH, and to investigate OCT-based vitreoretinal interface- and intraretinal abnormalities associated with MH formation.

## Methods

### Study design and participants

This retrospective, observational study was conducted at the Department of Ophthalmology at Stavanger University Hospital in Norway. Stavanger University Hospital is the only referral hospital for vitreoretinal surgery in Rogaland County and serves a population of approximately 450,000 inhabitants. As some residents in the northern part of the county may be referred elsewhere, we only included patients living in the 18 southern municipalities of Rogaland County. The medical records of 276 patients who underwent surgery for MH from January 2008 through December 2018 were reviewed. Inclusion criteria were primary MH with available OCT scans of the fellow eye from the time of primary MH diagnosis or surgery. One patient declined study participation and 46 patients were classified as having secondary MH. We categorised the patients into two groups: A bilateral group comprising subjects who subsequently developed MH in the fellow eye, and a unilateral group with subjects who did not develop MH in the fellow eye during follow-up. Macular OCT imaging was performed at the initial visit and the OCT features of the fellow eye in the two groups were compared.

The study was approved by the Regional Committee for Medical and Health Research Ethics (2018/954 REC west, Norway) and followed the tenets of the Declaration of Helsinki. Written informed consent was sent to all living patients, and the opportunity to decline study participation was offered.

### Background parameters and optical coherence tomography imaging

The following patient characteristics were retrieved from the electronic medical records: sex, date of birth, duration of symptoms, laterality, VA in logMAR, date of surgery, and date of death if deceased.

High-resolution OCT images were obtained using SD-OCT or SS-OCT (Topcon 3D OCT 2000 and Topcon DRI OCT Triton; Topcon Corp., Tokyo, Japan) of both eyes when the patient was examined for MH in the first eye. The scanning protocol used for SD-OCT was a macula 3D scan, 512 × 128 (6 × 6 mm, spacing 47 μm) centred on the macula, and for the SS-OCT a macula 3D scan, 512 × 256 (7 × 7 mm, spacing 23 μm) centred on the macula. The vitreomacular interface of the fellow eye was investigated and the PVD status categorised into the following stages:

I) No PVD: no signs of PVD; the posterior vitreous cortex attached to the retinal surface.
II) Perifoveal PVD: the posterior vitreous cortex attached to the fovea, but detached from the retinal surface around the fovea.
III) Foveal PVD: the posterior vitreous cortex not attached to the fovea, but attached to the optic disc. We classified cases where it was difficult to determine the relationship between the vitreous cortex and the optic disc as foveal PVD.
IV) Complete PVD: the posterior vitreous cortex detached from fovea and the optic disc.

**Fig. 1:**
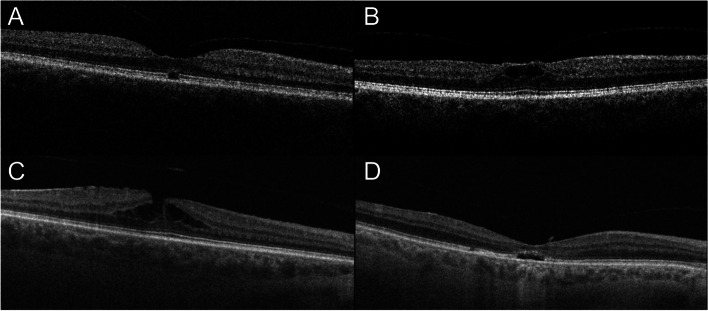
Optical coherence tomography scans illustrating intraretinal abnormalities we were looking for. **A** Outer retinal defect. **B** Intraretinal pseudocysts. **C** Intraretinal splits. **D** Foveolar detachment

Vitreomacular traction (VMT) was defined as the presence of anatomic distortion of the fovea in combination with perifoveal PVD, as described by Duker et al. [16]. The presence of other retinal abnormalities, such as intraretinal cysts, intraretinal splits, outer retinal defects (ORD), epiretinal membrane (ERM) and foveolar detachment in the central macular region was also registered (Fig. 1). Intraretinal splits were defined as tiny horizontal splits within the foveal region, and intraretinal cysts were defined as round-shaped intraretinal cavities [11, 17].

### Statistical analysis

Continuous data were described by mean and standard deviation (SD) when normally distributed, otherwise by median and range. Categorical data were summarised by numbers and proportions. The chi-square test or Fisher’s exact test was used for comparing categorical values. The Student’s t-test was used to compare normally distributed continuous data, such as age. We used the Wilcoxon signed-rank test to compare related samples. The Wilson score interval was used for estimating binomial proportion confidence intervals (CI). To control the false discovery rate at 0.05, we applied the Benjamini-Hochberg procedure. The statistical analyses and graphics were made using R Project for Statistical Computing, version 4.0.2 (R Foundation for Statistical Computing, Vienna, Austria). Two-tailed *p-*values ≤0.05 were considered statistically significant.

## Results

### Participants

**Fig. 2:**
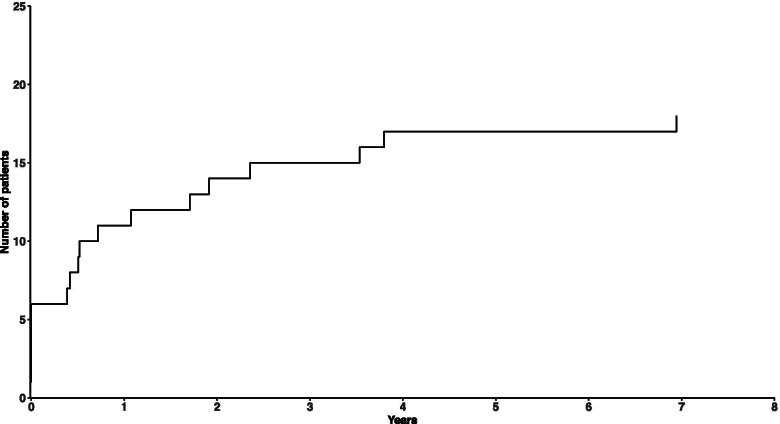
Cumulative frequency curve of macular hole in the fellow eye after diagnosis of macular hole in the first eye. Six patients who presented with bilateral macular hole at the initial visit were included

Between January 2008 and December 2018, 229 patients underwent surgery for primary MH. Twenty patients were identified with bilateral disease. Six of the patients presented with bilateral MH, of whom two had an old MH in the fellow eye unsuitable for surgery. Two patients had been operated for a MH in their fellow eye prior to 2008. A total of 12 patients subsequently developed a MH in their fellow eye and were enrolled in the bilateral group. Among the 209 patients with unilateral MH, 9 patients did not have an OCT image of their fellow eye at the initial examination and one patient was excluded due to poor OCT image quality. One patient was excluded due to a prosthesis in the fellow orbit, and one because of previous vitrectomy in the fellow eye. Hence, 197 patients were enrolled in the unilateral group. When calculating the overall risk of bilateral disease, we excluded the two patients with previous surgery in the fellow eye. The mean age at time of operation was 70.6 ± 8.6 years in the unilateral group and 71.7 ± 4.8 years in the bilateral group (*p* = 0.50, Student’s t-test). The male-to-female ratio in the bilateral group was 1:5 and 1:1.9 in the unilateral group (*p* = 0.35, Fisher’s exact test). Twenty patients presented with a history of MH or subsequently developed a MH in the fellow eye. Hence, the overall risk of bilaterality was 8.8% (95% CI, 5.8–13.2%). The risk of subsequent MH development was 5.7% (95% CI, 3.3–9.8%). Figure 2 illustrates the cumulative frequency of bilateral MH. The median observational time was 54 months (range, 3–138 months). In the bilateral group, the median time interval between the diagnosis of the first and the second MH was 17 months (range, 5–83 months), and 75% of the patients developed the MH in their fellow eye within 32 months. Two of the 12 patients in the bilateral group and 31 of the 197 patients in the unilateral group were pseudophakic in the fellow eye at baseline (*p =* 1.0, Fisher’s exact test). In the period until MH development in the fellow eye, two patients in the bilateral group underwent cataract surgery. Consequently, 8 of the 12 patients in the bilateral group were phakic at the time of MH formation in the relevant eye. Table 1 summarises the baseline demographics and OCT features of the two groups.

**Table 1:** Baseline demographics and optical coherence tomography features of the fellow eye

	Bilateral group (*n* = 12)	Unilateral group (*n* = 197)	*P*
Age, mean (SD), years	71.7 (4.8)	70.6 (8.6)	0.509*
Sex, male/female	2/10	68/129	0.345^†^
Pseudophakia, *n* (%)	2 (16.7)	31 (15.7)	1.0^†^
Interval between both eyes, median (range), months	17 (5–83)	NA	
Vitreoretinal relationship, *n* (%)			
No PVD	2 (16.7)	19 (9.6)	0.352^†^
Perifoveal PVD	7 (58.3)	81 (41.1)	
Foveal PVD	3 (25.0)	74 (37.6)	
Complete PVD	0	23 (11.7)	
Retinal abnormalities, *n* (%)			
VMT	3 (25.0)	33 (16.8)	0.438^†^
Epiretinal membrane	4 (33.3)	50 (25.4)	0.512^†^
Outer retinal defects	5 (41.7)	13 (6.6)	**0.001**^**†**^
Intraretinal splits	4 (33.3)	27 (13.7)	0.083^†^
Pseudocysts	4 (33.3)	20 (10.2)	0.036^†^
Foveolar detachment	0	6 (3.0)	1.0^†^

*MH* Macular hole, *NA* Not applicable, *PVD* Posterior vitreous detachment, *SD* Standard deviation, *VMT* Vitreomacular traction

* Students t-test.

^†^ Fisher’s exact test.

*p*-values that remain statistically significant after applying the Benjamini-Hochberg procedure for multiple testing are presented in bold.

### Optical coherence tomography findings

**Fig. 3:**
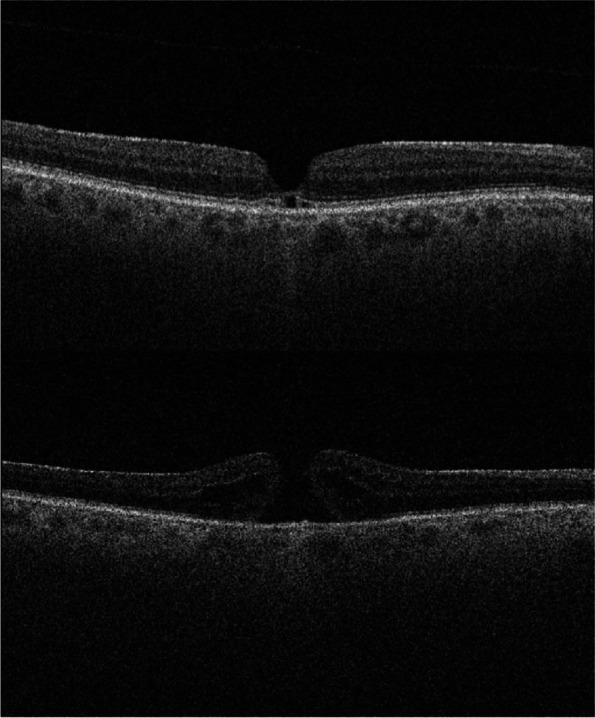
The upper image shows an optical coherence tomography scan of the fellow eye of a patient with macular hole at the initial visit. Outer retinal defects, foveal posterior vitreous detachment and a thin epiretinal membrane are present. The lower image, captured four months later, shows that the patient has developed a full-thickness macular hole

Foveal PVD in the fellow eye occurred in three patients (25%) in the bilateral group and in 74 patients (37.6%) in the unilateral group (*p* = 0.35). Figure 3 demonstrates the development of MH in a patient with foveal PVD. None of the patients in the bilateral group had a complete PVD compared to 23 patients (11.7%) in the unilateral group (*p* = 0.35). Although not significant, the extent of the PVD in the fellow eye seemed to be more advanced in the unilateral group compared to the bilateral group. The presence of VMT and ERM in the fellow eye was not significantly different between the two groups.

**Fig. 4:**
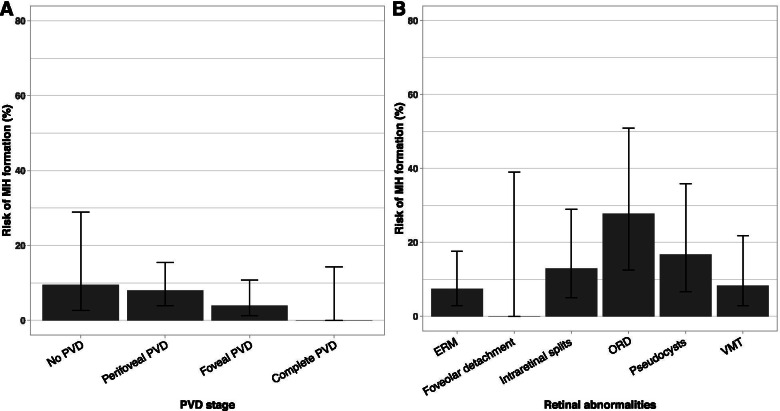
Bar graph illustrating the risk of subsequent MH formation in the fellow eye depending on **A** PVD stage and **B** the presence of retinal abnormalities. ERM = epiretinal membrane; MH = macular hole; PVD = posterior vitreous detachment; ORD = outer retinal defects; VMT = vitreomacular traction

Outer retinal defects were present in 41.7% of the fellow eyes in the bilateral group and in only 6.6% of the fellow eyes in the unilateral group (*p* = 0.001). The presence of ORD had a sensitivity of 41.7% (95% CI, 19.3–68.0%) and specificity of 93.4% (95% CI, 89.0–96.1%) in detecting subsequent MH formation. The presence of pseudocysts was also higher in the bilateral group with 33.3% compared to 10.2% in the unilateral group (*p* = 0.036), but did not remain statistically significant after correction for multiple testing. There were no statistically significant differences regarding the presence of intraretinal splits and foveolar detachment. All three patients in the bilateral group with foveal PVD in the fellow eye displayed ERM, ORD and a visible pseudo-operculum in the same eye. Among the patients with ORD in the fellow eye, 27.8% (95% CI, 12.5–50.9%) subsequently developed a MH. The presence of ORD was the strongest predictor of MH development in the fellow eye (Fig. 4). Table 2 shows a comparison of VA, MH diameter and duration of symptoms between the first and the second eye, for which no significant differences were found.

**Table 2:** Comparison of first and second eye in the bilateral group

	First eye	Second eye	*P*
Preoperative VA, mean (SD), logMAR	0.63 (0.14) (*n*=10)	0.63 (0.19) (*n*=12)	0.81*
MH diameter, mean (SD), μm	356 (177) (*n*=12)	388 (169) (*n*=12)	0.52*
Duration of symptoms, median (range), months	5 (1−8) (*n*=10)	4 (1−12) (*n*=12)	0.76*

*MH* Macular hole, *SD* Standard deviation, *VA* Visual acuity

* Wilcoxon signed-rank test

## Discussion

In this study, we found an overall risk of bilateral MH of 8.8%, illustrating a profoundly higher probability of MH in the fellow eye compared to the risk of first eye MH in the general population [1, 2]. The risk of subsequent MH formation was 5.7%. These results are in accordance with those of McCannel et al., Furashova & Matthè, and Lewis et al. [2, 4, 18]. Other studies however, have reported a higher risk of bilateral MH formation [7, 8]. Ezra et al. reported a bilateral risk of 15.6%, but their study was based on a subgroup of fellow eyes without PVD, which may have caused overestimation of the overall risk of bilateral MH formation [9]. Ali et al. reported that Asian-Americans had a 177% increased risk of MH formation compared to Caucasians [19]. Ethnic susceptibility to MH formation may partly explain why Kumagai et al. and Choi et al. reported a higher risk of bilateral MH formation [7, 8]. Our calculated risk may be underestimated as individuals in the unilateral group may develop a MH in the fellow eye after ended data collection. Our study relied on the high probability of patients developing a MH in their fellow eye being readmitted to the Stavanger University Hospital. Still, we cannot fully exclude the risk that some patients have moved out of our catchment area or been referred elsewhere with a MH in the fellow eye. We did not investigate the family history of MH in our patients. Kay et al. reported a significantly higher frequency of MH among family members of patients with bilateral MHs, which may indicate a genetic predisposition in some individuals [20].

Although not statistically significant, PVD had reached a more progressed stage in the unilateral group, and no fellow eye with complete PVD developed full-thickness MH. Several studies have demonstrated that complete PVD is negatively associated with the development of MH [7, 18]. Surprisingly, three eyes with foveal PVD subsequently progressed to a full-thickness MH. Hence, VMT is not the only contributor to MH development and foveal or complete PVD does not rule out the possibility of MH formation. However, the presence of ORD and a pseudo-operculum in these three cases indicate previous vitreomacular traction and a weakened foveal structure. In a study by Takahashi et al., five out of 16 patients with foveal PVD subsequently developed a MH [11]. Besirli & Johnson described two cases with foveal PVD who developed MH, where OCT imaging revealed foveal contour irregularities consistent with previous vitreomacular traction [21]. Peeling of the internal limiting membrane improves the closure rates after MH surgery, which indicates the presence of tangential traction forces on the retinal surface [22]. In a prospective study on 34 individuals with lamellar MH, Bottoni et al. detected two patients with PVD and concomitant ERM who subsequently developed full-thickness MH [23]. A post hoc evaluation of the three patients with foveal PVD and MH formation in our study revealed that two of the cases had a thick ERM and one had a thin ERM. Recently, Bringmann et al. described different modes of MH formation and emphasised that MH formation is caused by disruption of both Müller cell cones and the external limiting membrane [24]. A plausible theory explaining our three cases with foveal PVD and MH formation, is that initially, vitreomacular traction caused structural damage to the fovea. Subsequently, this vulnerability facilitated the formation of a full-thickness MH induced by tangential traction by the ERM on the retinal surface.

Outer retinal defects were significantly more frequent in the bilateral group. In accordance with Choi et al., we found the presence of ORD to have the highest positive predictive value of developing MH, with a sensitivity of 41.7% in predicting MH formation [7]. However, while Choi et al. reported a specificity of 100%, we found it to be 93.4%. In our study, five out of 18 patients with ORD developed a MH. In contrast, all five eyes with ORD in the study by Choi et al. developed a MH. Nevertheless, many of our patients in the unilateral group had retinal abnormalities in the fellow eye. This is in accordance with the findings of Chhablani et al. and Kumagai et al., reporting that retinal abnormalities and vitreofoveal interface changes are more common in fellow eyes of patients with MH than in a matched healthy population [15, 25].

In the bilateral group, we found no significant differences between the first and the second eye regarding preoperative VA, MH size or duration of symptoms. One would expect that patients would seek medical assistance at an earlier stage when suffering from a MH in their fellow eye. In Norway, patients need a referral from a health care professional to access specialised hospital departments, which may explain some of the delay from onset of symptoms to treatment.

The present study has several limitations including its retrospective design and a relatively small sample size. We only examined the OCT images of the fellow eye captured at the time when the first eye was examined for a MH. A longitudinal study design with repeated OCT examinations could have revealed other transient retinal abnormalities and vitreoretinal interface changes. OCT images were available for 94% of the fellow eyes in the unilateral group and for all of the fellow eyes that subsequently developed MH. Due to the retrospective study design, two different OCT systems, SS-OCT and SD-OCT, were used in the study. SS-OCT provides narrower spacing and better detection of deeper signals, posterior to the retinal pigment epithelium (RPE). However, both SS-OCT and SD-OCT use the Fourier domain detection techniques and allow detection of subtle retinal changes anterior to the RPE [26].

## Conclusion

Our study provides useful information when counselling patients with MH. This patient group has a substantially increased risk of developing a MH in the fellow eye compared to the general population. The presence of complete PVD indicates a minimal risk of developing a MH, while the presence of ORD reveals a significantly higher risk of MH formation.

## Data Availability

The dataset is available from the corresponding author on reasonable request.

## Abbreviations

CI: Confidence interval
ERM: Epiretinal membrane
MH: Macular hole
OCT: Optical coherence tomography
ORD: Outer retinal defects
PVD: Posterior vitreous detachment
RPE: Retinal pigment epithelium
SD: Standard deviation
SD-OCT: Spectral domain optical coherence tomography
SS-OCT: Swept source optical coherence tomography
VA: Visual acuity
VMT: Vitreomacular traction
